# Structured multifrequency segmental bioimpedance descriptors for diabetes screening support

**DOI:** 10.3389/fphys.2026.1958735

**Published:** 2026-10-29

**Authors:** Minho Choi, Kun Ho Lee, Jaeuk U. Kim

**Affiliations:** 1 Life Science Research Division, Korea Institute of Oriental Medicine, Daejeon, Republic of Korea; 2 Gwangju Alzheimer’s and Related Dementia Cohort Research Center, Chosun University, Gwangju, Republic of Korea; 3 Department of Biomedical Science, Chosun University, Gwangju, Republic of Korea; 4 Korea Brain Research Institute, Daegu, Republic of Korea; 5 KM Convergence Science, University of Science and Technology, Daejeon, Republic of Korea

**Keywords:** bioelectrical impedance analysis, diabetes mellitus, diabetes screening, frequency-interval descriptor, machine learning, multifrequency bioimpedance, reliability-weighted descriptor

## Abstract

**Background:**

Blood-based measurements remain the standard for identifying diabetes mellitus (DM), but repeatable non-invasive approaches may have value for screening support. Bioelectrical impedance analysis (BIA) captures body-composition, hydration, and bioelectrical properties associated with metabolic health. However, previous studies have largely focused on analyzer-derived body-composition variables or individual parameters such as phase angle, whereas multifrequency segmental impedance responses have not been systematically characterized for screening-support applications. This study aimed to computationally characterize multifrequency segmental BIA responses using structured descriptors and to evaluate their potential for identifying previously diagnosed DM status within a proof-of-concept screening-support framework.

**Methods:**

An ExtraTrees-based classification framework was developed to discriminate previously diagnosed DM status. Feature selection, input dimensionality determination, and model optimization were performed within a nested cross-validation framework, and performance was evaluated using pooled out-of-fold predictions. Alongside demographic and device-derived body-composition variables, we formulated 440 base BIA descriptors capturing amplitude and dispersion, phase angle, Cole and impedance geometry, curve shape and coupling, and regional-pattern information. We additionally developed frequency-interval and reliability-weighted interval descriptors to characterize interval-specific variations in multifrequency impedance-magnitude responses and their local agreement with the fitted frequency-response trend. Model performance was assessed using the area under the receiver operating characteristic curve (AUROC) and average precision (AP), together with calibration and threshold-dependent metrics.

**Results:**

Evaluated in 2,745 participants aged 47–90 years from a predominantly older-adult cohort, the ExtraTrees model achieved an AUROC of 0.8336 (95% CI, 0.8138–0.8523) and an AP of 0.6754 (95% CI, 0.6395–0.7092). The full descriptor configuration showed the highest overall discrimination, although the incremental gains associated with the newly developed frequency-interval and reliability-weighted descriptors were modest. These two descriptor groups together accounted for approximately 35.1% of the pooled out-of-fold SHAP contribution, consistent with a complementary rather than dominant predictive role.

**Conclusion:**

This study demonstrates the feasibility of using structured multifrequency segmental BIA-derived descriptors to discriminate previously diagnosed DM status. The newly developed descriptors provided complementary predictive information, although their incremental improvement in discrimination was limited. These findings provide a methodological basis for future evaluation of BIA-assisted screening using contemporaneous blood-test-defined outcomes in appropriately recruited populations.

## Introduction

Diabetes mellitus (DM) is a chronic metabolic disorder and a major contributor to microvascular and macrovascular complications (Kharroubi and Darwish, 2015; Ong et al., 2023). Its prevalence increases substantially with age, making early identification and appropriate management particularly important in older populations (Kirkman et al., 2012; Soriguer et al., 2012; Sinclair et al., 2015). Because DM may remain unrecognized until complications or metabolic abnormalities become clinically apparent, accessible approaches that can support repeated screening and guide individuals toward confirmatory testing may have practical value. However, definitive identification of DM continues to rely on blood-based measurements such as fasting plasma glucose and glycated hemoglobin (HbA1c) (American Diabetes Association Professional Practice Committee, 2026b).

Bioelectrical impedance analysis (BIA) is a non-invasive technique that characterizes body composition, hydration, and tissue bioelectrical properties. These characteristics are relevant to DM because metabolic alterations may be accompanied by changes in fat and muscle distribution, body-water balance, and cellular properties (Ward, 2019). Previous studies have therefore investigated BIA-derived parameters in relation to DM. Phase angle (PhA), which reflects cell membrane integrity, body cell mass, and body-water distribution, has been reported to differ between individuals with and without DM (Buscemi et al., 1998; Jun et al., 2021; Nandwani et al., 2025). Notably, Jun et al. demonstrated that maximum segmental PhA values could discriminate DM status in men and women, with area under the receiver operating characteristic curve (AUROC) values of 0.827 and 0.845, respectively (Jun et al., 2018). Beyond PhA, BIA-derived measures of visceral adiposity, fat-to-muscle ratio, and body-water distribution have also been associated with type 2 DM (Wang et al., 2021; Li et al., 2022; Lee et al., 2025).

Previous studies have reported associations between BIA and DM and have suggested the potential of BIA-based approaches for DM screening. However, many of these studies primarily focused on a limited number of BIA-derived variables, such as PhA, FMR, or body water distribution indices. Given that DM is associated with complex physiological alterations, including decreased muscle mass, visceral fat accumulation, abnormal body water distribution, and changes in cell membrane function, an integrated approach that considers bioelectrical response patterns across multiple body segments and frequencies may be needed rather than relying on single or limited indicators. Moreover, relatively few studies have systematically transformed these measurements into structured descriptors and evaluated their collective predictive utility within a machine-learning framework. Thus, it remains unclear whether more comprehensive characterization of multifrequency segmental bioimpedance responses can provide additional information for DM screening-support applications.

To address this gap, the present study makes three main contributions. First, we systematically organized and partially extended previously proposed BIA parameters into structured high-dimensional descriptor groups. Measured impedance and reactance data were analyzed from multiple perspectives and categorized into amplitude and dispersion, phase angle, Cole and impedance-geometry, curve-shape and coupling, and regional-pattern descriptors. These descriptors were intended to more comprehensively characterize bioelectrical response patterns potentially associated with DM across body segments and frequencies. Second, we newly constructed frequency-interval descriptors and reliability-weighted interval descriptors to characterize interval-specific changes in impedance magnitude, the overall frequency-response trend, and local deviations from the fitted curve. These descriptors were designed to provide additional information on frequency-dependent impedance-response patterns beyond conventional BIA parameters. Finally, we developed an extremely randomized trees (ExtraTrees)-based classification model using the constructed descriptors together with demographic and device-derived body-composition variables and evaluated its performance in 2,745 participants. Model performance was compared across descriptor combinations and input feature dimensions within a nested cross-validation framework, and classifier sensitivity and model calibration were additionally evaluated.

A BIA-based model should not be viewed as a replacement for conventional diagnostic testing. Rather, the present study evaluates whether structured BIA information associated with previously diagnosed DM can provide a methodological basis for future screening-support research using appropriately defined screening outcomes. Accordingly, this study aimed to computationally characterize multifrequency segmental bioimpedance responses using structured descriptors and to evaluate whether these descriptors could support discrimination of previously diagnosed DM status in a predominantly older-adult cohort. The study further examined the incremental predictive value of the newly constructed frequency-interval and reliability-weighted interval descriptors beyond demographic, device-derived body-composition, and base BIA-derived descriptors. This analysis was intended as a proof-of-concept evaluation of BIA-based DM screening support rather than as validation of a standalone diagnostic tool.

## Methods

### Study population and outcome definition

The data for this study were collected as part of a research project aimed at constructing a multi-biomedical database for dementia and developing early diagnostic prediction technologies. The present investigation was conducted as a secondary analysis of this cohort because standardized multifrequency segmental BIA measurements and medical-history information, including previously diagnosed DM status, were simultaneously available for a relatively large number of participants. All procedures in this study were approved by the Institutional Review Board of Chonnam National University Hospital (IRB No. CNUH-2019-279). In compliance with the relevant guidelines and regulations, data were collected from a total of 2,745 participants aged 47–90 years at the Gwangju Alzheimer’s and Related Dementias Cohort Research Center between 2019 and 2022. The cohort had a mean age in the early 70s and therefore predominantly comprised older adults. Before data collection, all participants received a detailed explanation of the study objectives and procedures, and written informed consent, which included the secondary use of data for health-related research, was obtained from each participant. All procedures were performed in accordance with the principles of the Declaration of Helsinki. The study collected information on participants’ demographic characteristics, current and past medical history, and other health-related variables. In addition, a comprehensive battery of clinical and physiological assessments was conducted, including body-composition measurements. In the present study, demographic, body-composition, and BIA data were used for DM classification. DM status was determined from self-reported history of previously diagnosed DM obtained during medical interviews and was used as the classification outcome. The outcome was not defined using contemporaneous blood glucose or HbA1c measurements and therefore did not identify undiagnosed DM or DM defined by current glycemic status. For descriptive characterization of the study population, continuous baseline variables were presented as mean ± standard deviation (SD) and compared between participants with and without previously diagnosed DM using Welch’s t-test (Ward et al., 2024). Sex distribution was compared using the chi-square test. These analyses were performed solely to characterize the study population and were not used for feature selection or model development.

### BIA measurement protocol

Body composition was assessed using the InBody S10 (InBody Co., Seoul, Republic of Korea), a high-performance professional body water analyzer (Alvero-Cruz et al., 2021). This device utilizes a tetrapolar 8-point tactile electrode system to measure bioelectrical impedance across six frequencies (1, 5, 50, 250, 500, and 1,000 kHz) and reactance across three frequencies (5, 50, and 250 kHz). Based on these measurements, various body composition indices are calculated for the total body as well as five specific segments: the right arm, left arm, trunk, right leg, and left leg. Although this equipment can accommodate participants in various positions, all measurements in this study were performed with the participants in a supine position. This was done to prevent gravity-induced shifts in body water and to minimize distortions in measurement values caused by subtle muscle tension or body movement.

### Descriptor construction

Conventional body composition analyzers measure impedance and reactance at various frequencies and calculate diverse body composition indices by integrating these data with information such as age, sex, height, and weight. Therefore, these four standard parameters commonly required by commercial body composition analyzers were defined as demographic variables (*D_d_*) and incorporated into the DM screening. Furthermore, the raw impedance and reactance values—measured across the five body segments at six and three frequencies, respectively—served as the baseline bioelectrical data. These raw values were subsequently reprocessed into high-dimensional descriptors designed to characterize bioelectrical patterns potentially relevant to DM screening. The specific methodology for constructing these descriptors is detailed in the subsequent sections. Along with these, the device-derived segmental body composition variables, denoted as *D_s_*, were also utilized; these included segment-specific body water, ECW/TBW, and lean mass generated by the analyzer.

The descriptors used in this study were organized into five groups: *D_d_*, *D_s_*, base BIA descriptors (*D_b_*), frequency-interval descriptors (*D_f_*), and reliability-weighted interval descriptors (*D_r_*). The detailed construction of the BIA-derived descriptor groups is described in the following subsections. For methodological clarity, the BIA-derived descriptor groups were also distinguished according to their methodological origin. The *D_b_* group comprised established BIA parameters together with descriptors adapted or extended from previously reported electrical, geometric, and regional concepts. In contrast, *D_f_* and *D_r_* were newly developed in the present study. *D_f_* was designed to characterize interval-specific changes in impedance magnitude across frequency, whereas *D_r_* incorporated local deviations from the fitted frequency-response trend to weight these interval-specific changes according to their consistency with the overall frequency-response pattern.

#### Base BIA descriptors

In addition to the raw values and arithmetic combinations of impedance and reactance for each segment and frequency, various BIA descriptors based on the electrical relationships and geometric information of impedance and reactance—such as PhA and Cole-related parameters—have been proposed in previous literature. In this study, these previously established or partially extended forms of descriptors were defined as *D_b_*. Specifically, *D_b_* encompasses the following five distinct categories: amplitude and dispersion descriptors, PhA descriptors, Cole and impedance-geometry descriptors, curve-shape and coupling descriptors, regional-pattern descriptors. The detailed list and specifications of all 440 individual descriptors within *D_b_* are provided in **Supplementary Table S1**.

Amplitude and dispersion descriptors summarized the magnitude and frequency-dependent dispersion of impedance and reactance. Descriptors in this category quantify statistical characteristics of impedance or reactance values, either for the whole body or for individual body segments, including the mean, SD, maximum, and minimum values. They also capture frequency-dependent variations, such as decreases or slopes across frequencies. These descriptors represent the overall level and frequency-dependent spread of bioelectrical responses associated with tissue composition, conductive pathways, and fluid distribution (Wagner, 2022; Company-Se et al., 2023, 2025; Serafini et al., 2025).

Impedance (*Z*) is a complex quantity composed of resistance (*R*) and reactance (*X*). The impedance values measured by the device in this study represent the magnitude of impedance ( $\left|Z\right|=\sqrt{R^{2}+X^{2}}$) (Kyle et al., 2004). PhA, calculated from resistance and reactance, is an established BIA parameter reflecting the phase relationship between the resistive and capacitive components of biological tissue (Jun et al., 2018; Franco-Oliva et al., 2023; Ward and Brantlov, 2023; Soler-Climent et al., 2026). In this study, both frequency-specific PhA values and descriptors summarizing their changes across frequencies were included as PhA descriptors.

Cole-related descriptors were derived from the geometric and frequency-dependent relationships between resistance and reactance on the *R*-*X* plane according to established Cole-model concepts (Cole and Cole, 1941; Khalil et al., 2014; Gholami-Boroujeny and Bolic, 2016). Because reactance was available at three frequencies in the present dataset, the corresponding three R-X points were used to derive the Cole and impedance-geometry descriptors (Fu and Freeborn, 2020; Reljin et al., 2020; Freeborn and Critcher, 2021).

Curve-shape and coupling descriptors were constructed to quantify changes in multifrequency BIA measurements across frequencies. Whereas amplitude and dispersion descriptors summarize the average levels and simple decreases in impedance or reactance, curve-shape descriptors regard the frequency-dependent responses of impedance and reactance as curves and aim to capture more detailed morphological information, including curvature, roughness, and peak-related characteristics (Kusche and Ryschka, 2021). In addition, coupling descriptors quantify the ratio or local relationship between reactance and resistance at matched frequencies. Thus, in the present study, curve-shape and coupling descriptors were designed to complementarily summarize the detailed morphology of multifrequency BIA measurements and the dynamic relationships between their electrical components, including subtle curve shapes, nonlinearity, local variations, and coupling patterns between reconstructed resistance and reactance.

Regional-pattern descriptors were designed to represent inter-segmental relationships and whole-body distribution patterns, including trunk–limb contrast, bilateral asymmetry, and pairwise segment similarity (D’Hondt et al., 2022; Wako et al., 2025). Although descriptors from other categories may also aggregate whole-body information derived from individual segments, the regional-pattern descriptors more directly and granularly capture the topographical variations across the five body segments. Specifically, they contextualize these localized bioelectrical differences to reflect the structural harmony, fluid shifting, or tissue heterogeneity inherent in the entire body composition.

#### Frequency-interval descriptors

Similar to the Cole and impedance-geometry descriptors of *D_b_*, a representative approach to analyzing frequency-dependent impedance responses involves plotting the measured values on the *R*–*X* plane and applying Cole or Cole-Cole model fitting. This approach simultaneously considers resistance and reactance, enabling the estimation and interpretation of biophysical parameters such as *R*_0_, *R*_∞_, *f_c_*, and *α* (Wang et al., 2023). In addition to this conventional method, the present study constructed an impedance fitting curve (IFC) by performing robust fitting on the profile of impedance magnitude changes across a log-frequency scale (**Figure 1**). The interval-specific variation characteristics derived from this curve were collectively defined as *D_f_*. This approach is distinguished from the curve-shape and coupling descriptors of *D_b_*, which are derived from raw impedance or reactance values and their local relationships. In contrast, *D_f_* focuses on detailed changes across frequency intervals using the IFC. Compared to *R*–*X* plane-based analysis, this methodology has certain limitations; because it relies solely on impedance magnitude, it cannot isolate the relative contributions of resistance and reactance, nor does it provide a direct complex impedance-based interpretation like Cole-type parameters. Nevertheless, the dataset utilized in this study provided reactance values at only three frequencies (5, 50, and 250 kHz), which posed constraints on performing a stable *R*–*X* plane-based Cole model fitting over the entire frequency range. Conversely, impedance magnitude values were available at six frequencies (1, 5, 50, 250, 500, and 1000 kHz). This comprehensive availability offered the advantage of delineating the curve geometry of the bioelectrical response over a broader frequency range, thereby allowing for the evaluation of variation patterns across different frequency intervals. Therefore, this study aimed to robustly summarize the frequency-dependent response as *D_f_* based on the IFC, which encompasses a greater number of frequency data points.

**Figure 1:**
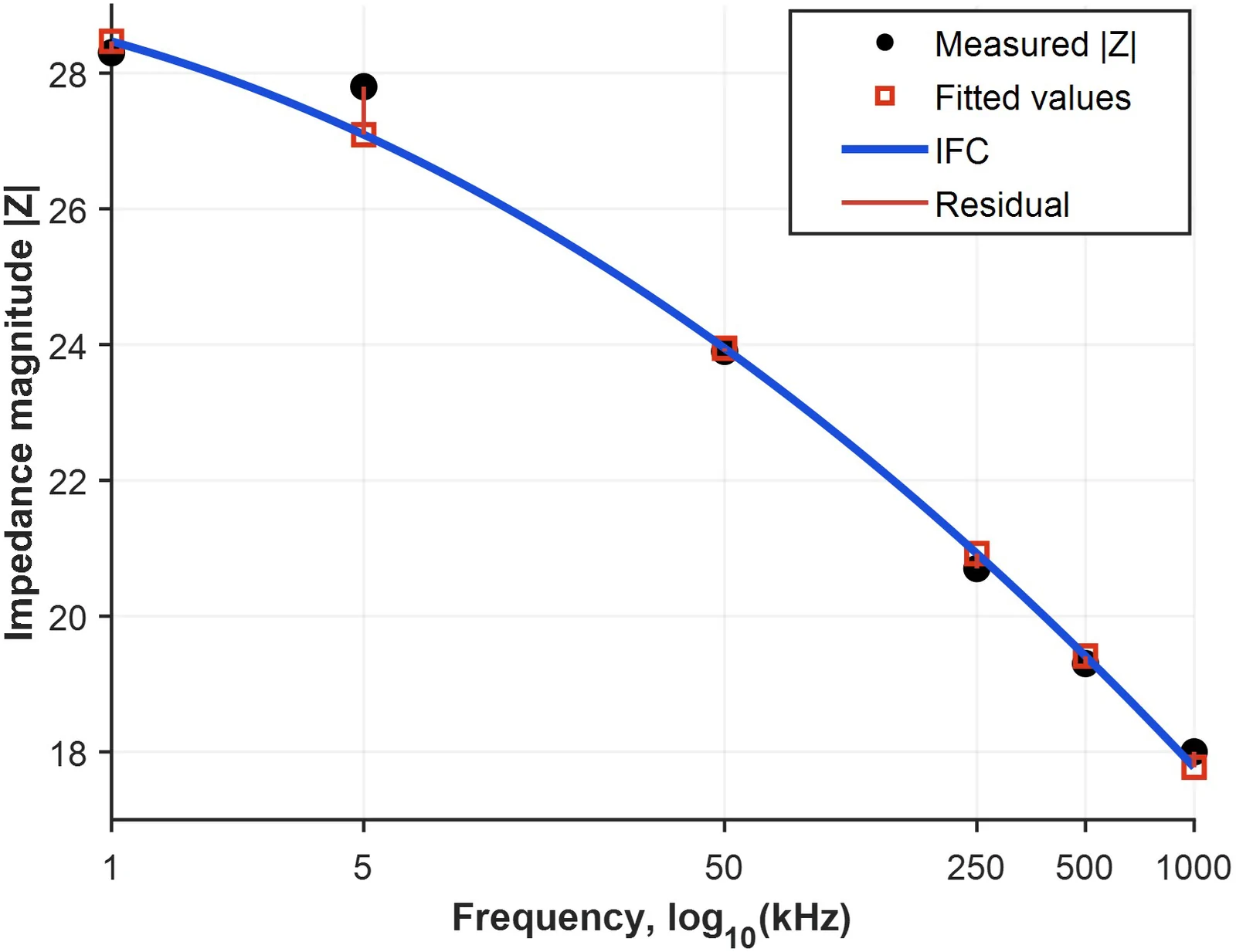
Example of IFC construction based on measured impedance values of the trunk.

Specifically, to calculate *D_f_*, the IFC was constructed separately for each body segment using the impedance-magnitude values measured at 1, 5, 50, 250, 500, and 1,000 kHz. Let $z_{i,j}=\left|Z_{i}\left(f_{j}\right)\right|$ denote the impedance magnitude for segment *i* at frequency *f_j_*, and let $x_{j}=\log_{10}\left[f_{j}/\left(1\text{ kHz}\right)\right]$. The IFC was represented by a quadratic function:


$${\hat{z}}_{i}\left(x\right)=a_{i}x^{2}+b_{i}x+c_{i},$$

where the coefficients were estimated using five iterations of robust iteratively reweighted least squares to reduce the influence of measurements that deviated substantially from the overall frequency-response trend. For each of the five adjacent frequency intervals, the slope (*S_i,j_*) and signed impedance drop (Δ*_i,j_*) were calculated from the fitted values as


$$S_{i,j}=\frac{{\hat{z}}_{i}\left(x_{j+1}\right)-{\hat{z}}_{i}\left(x_{j}\right)}{x_{j+1}-x_{j}},\;\Delta_{i,j}={\hat{z}}_{i}\left(x_{j}\right)-{\hat{z}}_{i}\left(x_{j+1}\right),\;j=1,\ldots ,5.$$

Additional descriptors summarized the fitted changes over the low- (1–50 kHz), intermediate- (50–250 kHz), and high-frequency (250–1,000 kHz) ranges, as well as the overall distribution of the interval slopes and drops. These descriptors were calculated for each of the five body segments and supplemented by across-segment summary, regional-average, and bilateral-asymmetry measures, yielding a total of 180 *D_f_* descriptors. Detailed definitions are provided in **Supplementary Table S1**.

#### Reliability-weighted interval descriptors

The IFC constructed for the frequency-interval analysis of impedance magnitude can be regarded as a smooth trend representing the overall frequency-dependent impedance response; however, discrepancies may occur between the actually measured data and the constructed IFC at specific frequencies (**Figure 1**). These residuals suggest potential influences from local measurement variability, noise, or transient fluctuations in bioelectrical signals. Therefore, the present study utilized *D_r_* alongside *D_f_* to incorporate this residual information. For each measured frequency, the fitting residual was defined as the difference between the measured impedance magnitude and the corresponding fitted IFC value:


$$e_{i,j}=z_{i,j}-{\hat{z}}_{i}\left(x_{j}\right).$$

For each adjacent frequency interval, a residual-based reliability weight (*q_i,j_*) was calculated from the absolute residuals at its two endpoints. The reliability-weighted slope ( ${\tilde{S}}_{i,j}$) and impedance drop ( ${\tilde{\Delta }}_{i,j}$) were then defined as


$$q_{i,j}=\frac{1}{1+\left|e_{i,j}\right|+\left|e_{i,j+1}\right|},\;{\tilde{S}}_{i,j}=q_{i,j}S_{i,j},\;{\tilde{\Delta }}_{i,j}=q_{i,j}\Delta_{i,j},\;j=1,\ldots ,5.$$

Thus, an interval with smaller endpoint residuals received a weight closer to 1, whereas an interval showing greater deviation from the overall frequency-response trend was assigned a smaller weight. For each body segment, *D_r_* included five reliability weights, five reliability-weighted slopes, and five reliability-weighted impedance drops. These 15 descriptors were calculated for all five body segments and supplemented by their means and SDs across the segments, yielding a total of 105 reliability-weighted interval descriptors. The *D_r_* descriptors were constructed based on these reliability values and their weighted forms of the interval-specific slopes and drops included in *D_f_*. This allowed the descriptors to reflect how stably the variations within a specific frequency interval align with the overall frequency-response trend. Thus, *D_r_* served to attenuate the influence of local measurement variability and emphasize more reliable frequency-response patterns based on the interval-level information derived from *D_f_*.* A* complete list of 105 *D_r_* descriptors can be found in **Supplementary Table S1**, along with *D_b_* and *D_f_*.

### Model development and evaluation

#### Descriptor configurations

To evaluate the feasibility of DM screening based on the constructed descriptors, we developed classification models that predicted DM status using the input descriptor sets. The descriptors considered in this study consisted of five groups, *D_d_*, *D_s_*, *D_b_*, *D_f_*, and *D_r_*, comprising a total of 744 descriptors. To construct an optimized input feature set for the classifier and to assess the usefulness of each descriptor group for DM screening, we compared classification performance across various combinations of descriptor groups. Starting from the combination of *D_d_*, *D_s_*, and *D_b_*, which represents descriptors commonly used in BIA research, the evaluation was further expanded by considering scenarios that included the newly proposed *D_f_* and *D_r_*. For each descriptor configuration, feature selection and model optimization were performed independently within the corresponding training data. In addition to the primary analysis in which the input dimensionality (ID) was optimized separately for each descriptor configuration, an additional analysis was performed using a fixed ID across the BIA-based descriptor configurations.

#### Internal validation and model optimization framework

The performance of each classifier was evaluated using a nested CV scheme to prevent data leakage and reduce evaluation bias. The entire dataset was partitioned into five outer folds. Within each outer iteration, four folds were used as the outer-training subset and the remaining fold was retained as an independent outer-test subset. All feature selection, ID determination, and hyperparameter optimization procedures were performed exclusively using the outer-training subset. Model development within each outer-training subset was performed sequentially in two stages. In Stage 1, a 5-fold stratified inner CV was used to perform feature ranking and determine the optimal ID. After the feature subset had been fixed, Stage 2 employed a separate stratified inner CV with randomized search to optimize classifier hyperparameters. Once the optimal hyperparameters had been identified, the classifier was retrained on the entire outer-training subset using the selected features and was subsequently evaluated on the corresponding outer-test fold. The outer-test participants were therefore completely excluded from feature ranking, ID selection, and hyperparameter optimization. This nested process was repeated across all five outer folds. For each participant, the predicted probability obtained when that participant belonged to the held-out outer-test fold was defined as an out-of-fold (OOF) prediction. Each participant therefore contributed exactly one OOF prediction generated by a model that had not been trained or optimized using that participant. The predictions from the five outer-test folds were subsequently pooled to form a complete OOF prediction vector for the study population, which was used for the primary discrimination and calibration analyses.

#### Feature selection and stability assessment

Because the descriptor space was high-dimensional and included potentially redundant variables, feature ranking was performed for each descriptor configuration. For the primary ExtraTrees classifier, ExtraTrees-based feature importance was used for this ranking. Based on these importance scores, candidate IDs were evaluated to examine the effect of feature-set size on model performance. For the *D_d_* + *D_s_* configuration, which comprised 19 candidate descriptors, IDs of 5, 10, 15, and 19 were considered, whereas IDs of 50, 100, 150, 200, 250, and 300 were considered for the higher-dimensional descriptor configurations. Within Stage 1 of each outer fold, ExtraTrees feature importance was calculated independently in each of the five inner-training folds. For each candidate ID, the highest-ranked features from the corresponding inner-training fold were used to train the model, and performance was evaluated on the associated inner-validation fold. The ID yielding the highest mean AUROC across the five inner folds was selected; average precision (AP) was used as a tie-breaker, followed by preference for the smaller ID when both AUROC and AP were identical. Feature importance was then averaged across the five inner folds, and the top-ranked features corresponding to the selected ID were retained for Stage 2 hyperparameter optimization.

To assess the stability of feature selection across the outer folds, the cross-fold selection frequency and rank variability of individual descriptors were additionally evaluated. Within each outer fold, ExtraTrees feature importance was calculated independently in each of the five inner-training folds and then averaged across the five folds for each descriptor. Descriptors were ranked in descending order of the resulting mean importance, and the top-ranked descriptors corresponding to the selected ID were retained. The selection frequency of each descriptor was defined as the number of outer folds in which it was retained in the selected feature subset (range, 0–5). For each descriptor, the median and interquartile range (IQR) of its feature-selection ranks were calculated using only the outer folds in which that descriptor was selected. A lower median rank therefore indicates a generally higher position during feature selection, whereas a smaller rank IQR indicates less variation in that position across outer folds. No rank was assigned for outer folds in which a descriptor was not selected.

#### Classifier training and sensitivity analysis

The resulting input feature space was high-dimensional, and the descriptor groups represented heterogeneous information, including demographic variables, body composition variables, and BIA-derived features. Furthermore, because the descriptors were constructed by analyzing measured impedance data from multiple perspectives, including curve fitting and frequency-dependent response analysis, the input data were expected to contain nonlinear relationships with the output and substantial interdependence among variables. Considering these data characteristics, ExtraTrees was selected as the primary classifier in this study (Yousefi et al., 2024). ExtraTrees is a tree-based ensemble method similar to random forest (RF), which is widely used in machine learning; however, it effectively mitigates the risk of overfitting by introducing a higher degree of randomness during model training. In addition, ExtraTrees does not require explicit feature scaling, making it suitable for handling heterogeneous features, and can flexibly model nonlinear decision boundaries. Furthermore, by introducing strong randomness into both feature selection and split-threshold determination, ExtraTrees can reduce dependence on specific variables and improve model robustness, even when many correlated or partially redundant predictors are present.

In Stage 2, ExtraTrees hyperparameters were optimized using randomized search within the stratified inner CV after the feature subset had been fixed. Twenty-five candidate hyperparameter combinations were evaluated using AUROC as the optimization criterion. The best-performing hyperparameter combination was then used to refit ExtraTrees on the complete outer-training subset before evaluation on the corresponding outer-test fold. To assess the sensitivity of model performance to classifier choice, the full descriptor pool was additionally evaluated using RF, extreme gradient boosting (XGBoost), light gradient boosting machine (LightGBM), and elastic-net logistic regression (ELR) (Chen et al., 2025). Because use of an ExtraTrees-derived feature subset for all classifiers could favor the primary classifier, feature ranking and ID selection were repeated independently for each alternative classifier using the same nested two-stage framework. RF, XGBoost, and LightGBM used their respective model-derived feature importance scores, whereas ELR used the absolute standardized elastic-net coefficients. Candidate IDs of 50, 100, 150, 200, 250, and 300 were evaluated independently for each classifier. Hyperparameters for each classifier were optimized using randomized search within the training data. The corresponding search spaces are provided in **Supplementary Table S2**.

#### Performance evaluation

The primary performance metrics were the AUROC and AP, the latter of which enables a fairer evaluation of positive-class detection performance under class imbalance (Saito and Rehmsmeier, 2015). Point estimates of AUROC and AP were calculated from the pooled OOF predicted probabilities. Their 95% confidence intervals (CIs) were estimated using 2,000 participant-level bootstrap resamples of the paired observed outcomes and OOF predicted probabilities, with the 2.5th and 97.5th percentiles of the bootstrap distributions used as the confidence limits. To quantify differences between descriptor configurations, paired differences in AUROC and AP were calculated from the corresponding OOF predictions. Because each descriptor configuration generated one prediction for the same participant, bootstrap resampling was performed at the participant level while preserving the paired predictions across configurations. This allowed the uncertainty of the difference in discrimination performance to be estimated while accounting for the paired evaluation structure.

Calibration performance was additionally evaluated using the Brier score (BS), calibration intercept (CAI), and calibration slope (CAS) (Steyerberg et al., 2010). BS was calculated as the mean squared difference between the predicted probability and the observed binary outcome, with smaller values indicating lower overall probabilistic prediction error. CAI and CAS were estimated by fitting a logistic calibration model in which the observed outcome was regressed on the logit of the pooled OOF predicted probability. A CAI of 0 and a CAS of 1 represent ideal calibration. CAI reflects systematic over- or underprediction, whereas CAS reflects whether the predicted probabilities are overly or insufficiently dispersed. Calibration curves were additionally constructed from the pooled OOF predictions using 10 quantile-based probability bins.

As additional evaluation metrics, balanced accuracy (BACC), F1-score, precision, sensitivity, and specificity were calculated to evaluate the performance of the final classification model. Because these metrics are threshold-dependent, performance was evaluated not only at the default decision threshold of 0.5 but also at the optimal decision thresholds that maximized BACC and F1-score, respectively. The BACC- and F1-optimized thresholds were determined separately within each outer-training subset to avoid using outer-test outcomes for threshold selection. Specifically, OOF probabilities were generated within the outer-training subset using a stratified inner CV with the optimized model, and the thresholds maximizing BACC and F1-score were identified from these inner-CV predictions. The selected thresholds were then applied without modification to the corresponding held-out outer-test fold. Threshold-dependent performance metrics were calculated after pooling the resulting classifications across all five outer-test folds.

#### Model interpretation using SHAP

To examine the predictive contribution of individual descriptors and descriptor groups in the final ExtraTrees model, SHapley Additive exPlanations (SHAP) values were calculated for participants in each held-out outer-test fold (Lundberg et al., 2020). SHAP values were therefore obtained exclusively from participants who had not been used for feature selection, hyperparameter optimization, or model fitting in the corresponding outer iteration. Because the selected feature subset differed across outer folds, the fold-specific SHAP values were mapped back to a common 744-descriptor feature space. Features not selected in a particular outer fold were assigned a SHAP value of zero for participants in that fold. For each descriptor, overall predictive importance was quantified as the mean absolute zero-padded OOF SHAP value across all participants. Descriptors were ordered in descending order of this value and assigned sequential global SHAP-based importance ranks. For the 100 highest-ranked descriptors reported in **Supplementary Table S3**, the importance score ratio was calculated by normalizing each descriptor’s mean absolute OOF SHAP value to the sum of the corresponding values across these top 100 descriptors. Thus, the importance score ratios sum to 100% within the top 100 descriptors and should not be interpreted as proportions of the total SHAP importance across all 744 descriptors. The global SHAP-based importance rank was distinct from the fold-specific ExtraTrees feature-selection ranks used for the stability analysis described in Section 2.4.3; the former reflects pooled OOF predictive attribution, whereas the latter reflects relative importance during feature selection within each outer-training fold. At the descriptor-group level, two complementary summaries were calculated. First, the relative absolute SHAP contribution of each descriptor group was calculated separately within each outer fold and summarized across the five folds as the fold-averaged relative SHAP contribution (FAS). Second, all zero-padded outer-test SHAP values were pooled across participants in the common feature space, and each group contribution was normalized by the total absolute SHAP contribution to obtain the zero-padded pooled OOF relative SHAP contribution (POS). FAS therefore summarizes the consistency of relative group contribution across outer folds, whereas POS summarizes the overall predictive attribution after pooling all OOF participants. SHAP values were interpreted as model-specific predictive attributions and not as causal effects.

#### Computational implementation

All model-development analyses used a fixed random seed for reproducibility. For the primary ExtraTrees analysis, the nested framework comprised five outer folds, five inner folds for feature ranking and dimensionality selection, and a separate four-fold inner cross-validation for hyperparameter optimization, with 25 randomized hyperparameter combinations evaluated in each outer fold. The primary full-configuration ExtraTrees analysis involved approximately 700 classifier fits in total. Additional descriptor-comparison and alternative-classifier analyses followed the same general nested-CV framework, with feature selection and hyperparameter optimization performed independently within the corresponding training data. All computations were performed under 64-bit Microsoft Windows 11 Enterprise using Python 3.13.5 on an 18-core Intel Core i9-9980XE CPU at 3.00 GHz with 128 GB of RAM. No GPU acceleration was used. After feature selection and hyperparameter optimization, refitting the fold-specific ExtraTrees model on each outer-training set required a median of 2.03 s, based on five repeated timing measurements for each of the five outer folds.

## Results

### Characteristics of the study population

Among the 2,745 subjects in this study, 557 (approximately 20.29%) were identified as DM-positive. The baseline characteristics of the overall study population by group are summarized in **Table 1**. All four demographic variables showed statistically significant differences between the DM-positive and DM-negative groups, with p-values less than 0.001. Specifically, the mean age was higher in the positive group than in the negative group; however, the mean difference was relatively small at 1.34 years, considering the overall age range. For sex, the only categorical variable, women accounted for a larger proportion of the DM-negative group, with men comprising 39.53% of this group. In contrast, men accounted for 52.96% of the DM-positive group, slightly exceeding the proportion of women. Both height and body weight were higher in the DM-positive group, with mean differences of 1.68 cm and 1.80 kg, respectively.

**Table 1:** Demographic characteristics of positive and negative groups.

Variable	Negative,	Positive,	p-value
	N=2,188	N=557	
Age (years)	72.12 ± 6.33	73.46 ± 5.88	< 0.001
Sex (male/female)	865/1323	295/262	< 0.001
Height (cm)	158.57 ± 8.13	160.25 ± 8.24	< 0.001
Weight (kg)	62.08 ± 9.52	63.88 ± 9.52	< 0.001

### Stability and characteristics of the reduced descriptor representation

To examine whether the proposed structured descriptor framework yielded a reproducible reduced representation rather than a fold-specific collection of features, we first evaluated feature-selection characteristics across the five outer folds. The complete descriptor space comprised 744 candidate descriptors from five groups (*D_d_*, *D_s_*, *D_b_*, *D_f_*, and *D_r_*). For the full descriptor configuration examined in this section, candidate input dimensionalities of 50, 100, 150, 200, 250, and 300 were evaluated within each outer-training set, and the selected dimensionality averaged 210 descriptors across the five outer folds. Thus, approximately 28.2% of the original 744-descriptor space was retained on average. Both dimensionality selection and identification of the retained descriptors were performed exclusively within the corresponding outer-training data. We therefore examined whether this reduced representation was reproducible across outer folds at both the individual-descriptor and descriptor-group levels.

At the individual-descriptor level, feature-selection stability was evaluated using cross-fold selection frequency and variability in fold-specific feature-selection ranks. To summarize stability among the descriptors with the greatest overall predictive attribution, we focused on the 100 descriptors with the highest global SHAP-based importance, as reported in **Supplementary Table S3**. Global SHAP-based importance was calculated from pooled OOF SHAP values as described in Section 2.4.6. Among these 100 descriptors, 80 were selected in all five outer folds and 95 were selected in at least four of the five folds, whereas the remaining five were selected in three folds. A similar pattern was observed for the newly developed descriptor groups. Among the 18 *D_f_* descriptors included in the top 100, 15 were selected in all five outer folds and the remaining three were selected in four folds. Among the 14 *D_r_* descriptors, 12 were selected in all five folds, one was selected in four folds, and one was selected in three folds. Thus, among the 32 *D_f_* and *D_r_* descriptors in the top 100, 27 were selected in all five outer folds, four were selected in four folds, and one was selected in three folds. However, the median feature-selection ranks and their IQRs varied across individual descriptors, indicating that repeated inclusion in the selected feature set did not necessarily imply a fixed relative importance ordering across folds. The 15 *D_f_* and *D_r_* descriptors with the highest global SHAP-based importance are summarized in **Table 2** together with their cross-fold selection frequency and feature-selection rank variability. Fourteen of these 15 descriptors were selected in all five outer folds, whereas the remaining descriptor was selected in four folds. Overall, these findings indicate substantial reproducibility in the inclusion of highly ranked descriptors across outer folds, although their exact relative ranking was less stable.

**Table 2:** Stability characteristics of the 15 highest-ranked D_f_ and D_r_ descriptors.

Overall rank	Group	Descriptor	Selection frequency	Median rank	Rank IQR
3	*D_f_*	trunk_int_slope_std	5	11.0	15.0
17	*D_f_*	trunk_int_slope_min	5	41.0	48.0
21	*D_f_*	trunk_band_high_drop	5	49.0	38.0
22	*D_f_*	trunk_int_drop_5	5	41.0	93.0
26	*D_f_*	trunk_int_slope_4	5	42.0	31.0
27	*D_f_*	trunk_int_slope_5	5	68.0	62.0
29	*D_f_*	trunk_band_mid_dominance	5	54.0	94.0
33	*D_f_*	trunk_int_drop_4	4	46.5	62.0
34	*D_f_*	allseg_int_slope_mean_mean	5	113.0	99.0
38	*D_r_*	trunk_int_weighted_slope_2	5	57.0	102.0
44	*D_r_*	allseg_int_rel_5_std	5	38.0	32.0
49	*D_r_*	trunk_int_weighted_drop_4	5	103.0	15.0
50	*D_f_*	arm_lr_slope_i1	5	35.0	48.0
51	*D_r_*	trunk_int_weighted_slope_4	5	87.0	19.0
52	*D_r_*	right_arm_int_weighted_slope_5	5	26.0	4.0

At the descriptor-group level, the composition of the reduced feature sets was examined across the five outer folds and is summarized in **Table 3**. In the table, *N_t_* and *N_s_* denote the total number of descriptors in each group and the mean number of descriptors selected across the five outer folds, respectively. Additionally, *r_g_* and *r_f_* represent the proportion of selected descriptors within the group and the proportion of descriptors from the corresponding group within the fold-specific selected feature set, respectively. The values of *N_s_*, *r_g_*, and *r_f_* are presented as mean ± SD across the five outer cross-validation folds. According to the results, *D_d_* and *D_s_* exhibited higher *r_g_* values compared to the other groups, with mean values of 0.900 and 0.813, respectively. However, their overall proportions within the selected feature sets remained small due to the initially small *N_t_*. *D_b_* accounted for the largest proportion among the groups, with an *r_f_* of 0.522 ± 0.017. The corresponding *r_f_* values were 0.244 ± 0.026 for *D_f_* and 0.150 ± 0.019 for *D_r_*, indicating that the two newly developed descriptor groups together accounted for approximately 39.4% of the fold-specific selected feature sets. However, *D_f_* and *D_r_* together comprised 285 of the 744 candidate descriptors (38.3%). Thus, their 39.4% representation in the fold-specific selected feature sets was broadly proportional to their availability in the candidate descriptor pool rather than indicating preferential selection.

**Table 3:** Group-wise selection characteristics and SHAP-based predictive contributions of descriptors across outer cross-validation folds.

Descriptor	*N_t_*	*N_s_*	*r_g_*	*r_f_*	FAS	POS
*D_d_*	4	3.6 ± 0.5	0.900 ± 0.137	0.019 ± 0.007	0.055 ± 0.015	0.055
*D_s_*	15	12.2 ± 1.6	0.813 ± 0.110	0.065 ± 0.022	0.058 ± 0.017	0.058
*D_b_*	440	110.0 ± 43.3	0.250 ± 0.098	0.522 ± 0.017	0.536 ± 0.017	0.536
*D_f_*	180	52.8 ± 24.1	0.293 ± 0.134	0.244 ± 0.026	0.221 ± 0.017	0.221
*D_r_*	105	31.4 ± 13.2	0.299 ± 0.126	0.150 ± 0.019	0.130 ± 0.016	0.130

### Incremental information provided by the newly developed descriptors

After characterizing the cross-fold reproducibility and composition of the reduced descriptor representation, we next evaluated whether the newly developed *D_f_* and *D_r_* descriptors provided additional predictive information beyond the demographic, device-derived body-composition, and base BIA descriptors. Classification performance was therefore compared across different descriptor combinations, as summarized in **Table 4**. For each descriptor configuration, feature selection and input dimensionality were independently determined within the corresponding outer-training data through inner cross-validation. For the *D_d_* + *D_s_* configuration, candidate IDs of 5, 10, 15, and 19 were evaluated because only 19 descriptors were available, whereas candidate IDs of 50–300 were evaluated for the higher-dimensional configurations. Accordingly, the ID values presented in **Table 4** represent the mean of the selected IDs across the five outer folds. For *D_d_* + *D_s_*, an ID of 10 was selected in three outer folds and an ID of 19 in two folds, yielding a mean selected ID of 13.6. Even using only demographic information commonly employed in body composition analysis alongside device-derived segmental parameters—such as body water, ECW/TBW, and lean mass—the model achieved an AUROC of 0.8050 (95% CI, 0.7850–0.8243) and an AP of 0.6353 (95% CI, 0.5995–0.6705). The *D_b_*, constructed by processing raw impedance and reactance values, includes 440 descriptors to capture various aspects of BIA. When added to *D_d_* + *D_s_*, *D_b_* improved the AUROC and AP by 0.0192 and 0.0270, respectively. When *D_f_* and *D_r_* were individually added to *D_d_* + *D_s_* + *D_b_*, the resulting AUROC and AP values were 0.8289 and 0.6673 for *D_f_*, and 0.8252 and 0.6615 for *D_r_*, respectively. The full descriptor configuration, *D_d_* + *D_s_* + *D_b_* + *D_f_* + *D_r_*, achieved the highest performance among the evaluated configurations, with an AUROC of 0.8336 (95% CI, 0.8138–0.8523) and an AP of 0.6754 (95% CI, 0.6395–0.7092).

**Table 4:** DM classification performance according to input descriptor configuration.

Input descriptors	ID	AUROC (95% CI)	AP (95% CI)	BS	CAI	CAS
*D_d_* + *D_s_*	13.6	0.8050 (0.7850, 0.8243)	0.6353 (0.5995, 0.6705)	0.1108	-0.6107	0.5877
*D_d_* + *D_s_* + *D_b_*	200	0.8242 (0.8049, 0.8421)	0.6623 (0.6266, 0.6945)	0.1061	-0.0996	1.0606
*D_d_* + *D_s_* + *D_b_* + *D_f_*	190	0.8289 (0.8101, 0.8478)	0.6673 (0.6318, 0.7013)	0.1044	-0.0859	1.0138
*D_d_* + *D_s_* + *D_b_* + *D_r_*	280	0.8252 (0.8066, 0.8436)	0.6615 (0.6269, 0.6958)	0.1057	-0.0596	1.0752
*D_d_* + *D_s_* + *D_b_* + *D_f_* + *D_r_*	210	0.8336 (0.8138, 0.8523)	0.6754 (0.6395, 0.7092)	0.1036	-0.1034	0.9806

To further evaluate whether these differences exceeded the variability expected from model evaluation, the full descriptor configuration was compared with the other descriptor configurations using paired differences in AUROC and AP (**Table 5**). Compared with *D_d_* + *D_s_*, the full configuration showed increases of 0.0286 in AUROC (95% CI, 0.0148–0.0426) and 0.0401 in AP (95% CI, 0.0208–0.0597). Relative to *D_d_* + *D_s_* + *D_b_*, the corresponding differences were 0.0094 (95% CI, 0.0026–0.0164) and 0.0131 (95% CI, 0.0024–0.0239). In comparison with *D_d_* + *D_s_* + *D_b_* + *D_f_*, the differences were smaller, and the 95% CIs included zero for both AUROC and AP. In contrast, comparison with *D_d_* + *D_s_* + *D_b_* + *D_r_* yielded positive differences of 0.0084 (95% CI, 0.0022–0.0144) for AUROC and 0.0138 (95% CI, 0.0046–0.0231) for AP. These results indicate that the incremental improvements associated with the additional descriptor groups were generally modest, although positive differences were observed for several comparisons.

**Table 5:** Differences in AUROC and AP between the full descriptor set (D_d_ + D_s_ + D_b_ + D_f_ + D_r_) and other descriptor configurations.

Comparison	ΔAUROC (95% CI)	ΔAP (95% CI)
*D_d_* + *D_s_*	0.0286 (0.0148, 0.0426)	0.0401 (0.0208, 0.0597)
*D_d_* + *D_s_* + *D_b_*	0.0094 (0.0026, 0.0164)	0.0131 (0.0024, 0.0239)
*D_d_* + *D_s_* + *D_b_* + *D_f_*	0.0047 (-0.0015, 0.0105)	0.0081 (-0.0015, 0.0177)
*D_d_* + *D_s_* + *D_b_* + *D_r_*	0.0084 (0.0022, 0.0144)	0.0138 (0.0046, 0.0231)

Because the incremental value of the descriptor groups may be affected by differences in the number of selected features, an additional comparison was performed using the same feature dimensionality of 250 across the BIA-based descriptor configurations (**Table 6**). An ID of 250 was selected as a common dimensionality because the mean IDs of the corresponding descriptor configurations in **Table 4** were concentrated around the 200–300 range, making 250 a representative intermediate value for a fixed-dimensionality comparison. Under this fixed-dimensionality condition, the *D_d_* + *D_s_* + *D_b_* configuration achieved an AUROC of 0.8277 and an AP of 0.6680. The corresponding values were 0.8287 and 0.6741 after adding *D_f_*, and 0.8280 and 0.6696 after adding *D_r_*. The full descriptor configuration again showed the highest performance, with an AUROC of 0.8335 and an AP of 0.6756. Thus, the overall performance pattern was broadly maintained when the number of selected features was held constant, although the absolute differences among the descriptor configurations remained small. Taken together, these analyses indicate modest incremental predictive information from the newly developed descriptors beyond the base descriptor configuration, although the magnitude of improvement was small and was not supported equally across all pairwise comparisons.

**Table 6:** DM classification performance across descriptor configurations using 250 features.

Input descriptors	AUROC (95% CI)	AP (95% CI)	BS	CAI	CAS
*D_d_* + *D_s_* + *D_b_*	0.8277 (0.8081, 0.8466)	0.6680 (0.6331, 0.7027)	0.1051	-0.1552	0.9714
*D_d_* + *D_s_* + *D_b_* + *D_f_*	0.8287 (0.8099, 0.8470)	0.6741 (0.6392, 0.7070)	0.1041	-0.0753	1.0503
*D_d_* + *D_s_* + *D_b_* + *D_r_*	0.8280 (0.8090, 0.8468)	0.6696 (0.6360, 0.7023)	0.1049	-0.0439	1.0917
*D_d_* + *D_s_* + *D_b_* + *D_f_* + *D_r_*	0.8335 (0.8157, 0.8516)	0.6756 (0.6416, 0.7089)	0.1034	-0.1368	0.9396

### Predictive contributions and bioelectrical characteristics of stable descriptors

To further characterize how the reduced descriptor representation contributed to model predictions, SHAP-based predictive attribution was examined at both the descriptor-group and individual-descriptor levels. Group-level SHAP-based predictive contributions are summarized in **Table 3**. FAS represents the fold-averaged relative SHAP contribution and is presented as mean ± SD across the five outer folds, whereas POS represents a single relative contribution estimated from the pooled OOF SHAP values. *D_b_* showed the largest SHAP-based contribution, with an FAS of 0.536 ± 0.017 and a POS of 0.536. This was followed by *D_f_* (FAS, 0.221 ± 0.017; POS, 0.221) and *D_r_* (FAS, 0.130 ± 0.016; POS, 0.130). The contributions of *D_d_* and *D_s_* were comparatively smaller, with POS values of 0.055 and 0.058, respectively. Thus, *D_f_* and *D_r_* together accounted for approximately 35.1% of the pooled OOF SHAP contribution. This proportion did not exceed their representation in the candidate descriptor pool and therefore does not indicate disproportionate group-level predictive importance. Rather, the SHAP results show that descriptors from these groups participated in the multivariable prediction structure alongside the other descriptor groups.

At the individual-descriptor level, **Figure 2** presents the SHAP summary plot for the 20 highest-ranked descriptors based on pooled OOF SHAP values, with concise descriptor labels shown on the y-axis to indicate the corresponding feature and, where applicable, its body region and measurement frequency or frequency interval. The contributions were distributed across multiple descriptors rather than being concentrated in a single feature, and individual descriptors showed both positive and negative contributions to the model predictions across participants. The highest-ranked descriptors overall also spanned multiple bioelectrical dimensions, including PhA, Cole/impedance-geometry, reactance- and impedance-curve, regional-pattern, and frequency-interval characteristics, indicating that predictive attribution was not confined to a single type of BIA information. Detailed information on the 100 highest-ranked descriptors, including their SHAP-based importance and cross-fold feature-selection stability, is provided in **Supplementary Table S3**. The highly ranked *D_f_* and *D_r_* descriptors also showed distinct anatomical and frequency-related patterns. Among the 15 highest-ranked *D_f_* and *D_r_* descriptors summarized in **Table 2**, 11 were derived from the trunk, two represented across-segment summaries, and two reflected arm-related information. Nine of the 15 descriptors directly characterized impedance-response patterns within the 250–1,000 kHz range, including 250–500 kHz, 500–1,000 kHz, or the predefined high-frequency band. Most of these descriptors quantified interval-specific slopes or impedance drops, including their reliability-weighted forms. Thus, the repeatedly retained newly developed descriptors were predominantly associated with trunk-based and higher-frequency impedance-response patterns and primarily characterized the shape and consistency of frequency-dependent changes rather than absolute impedance magnitude alone.

**Figure 2:**
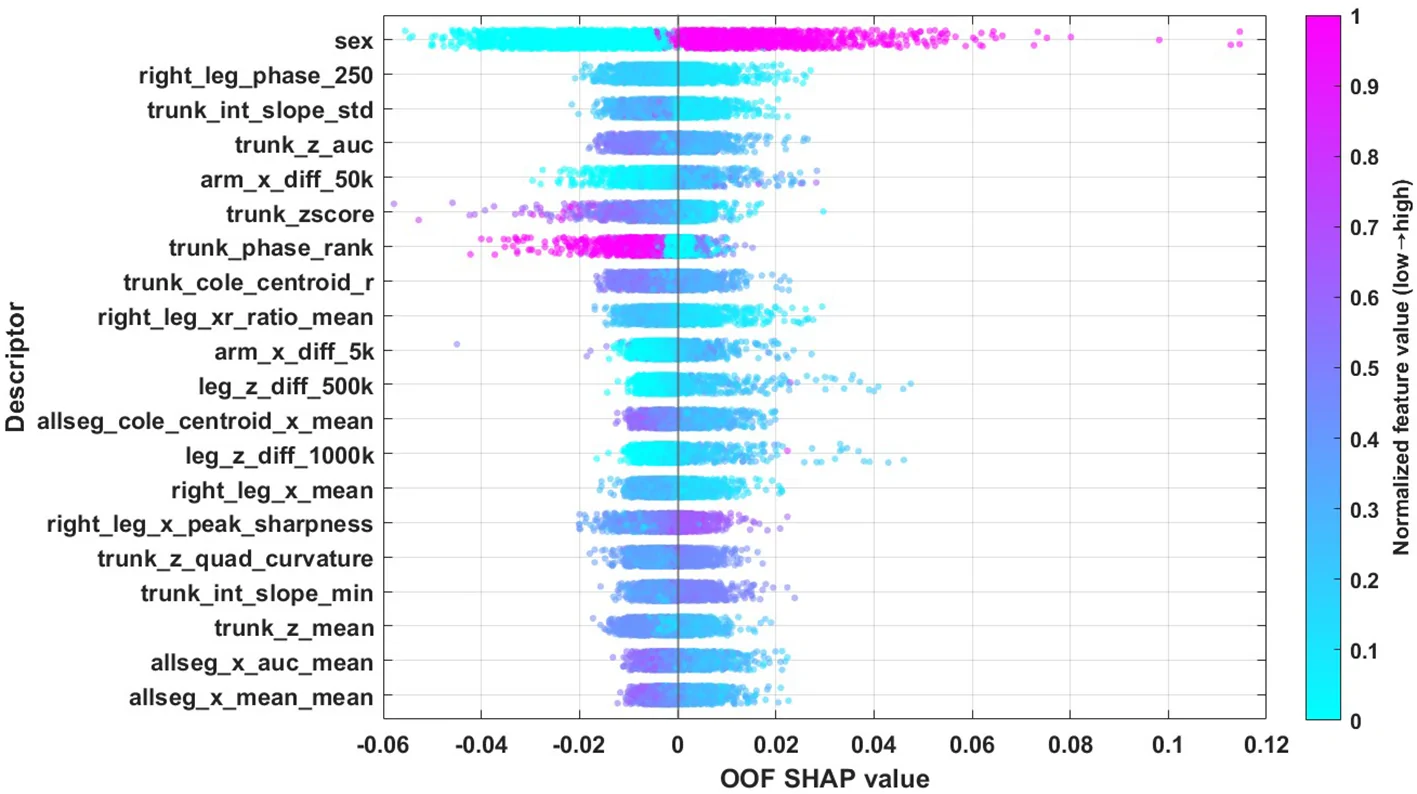
SHAP summary plot of the 20 highest-ranked descriptors in the final ExtraTrees model. Concise descriptor labels are shown on the y-axis, with detailed definitions provided in **Supplementary Tables S3**.

### Performance of the primary ExtraTrees classifier

Using pooled OOF predictions from the nested 5-fold CV, the ExtraTrees classifier achieved an AUROC of 0.8336 (95% CI, 0.8138–0.8523) and an AP of 0.6754 (95% CI, 0.6395–0.7092) (**Table 7**). In terms of AUROC, the model demonstrated moderate-to-good performance in discriminating DM across the outer-test folds (**Figure 3**). The fold-specific AUROC values ranged from 0.797 to 0.866, indicating generally consistent discrimination across the five validation folds. Calibration of the final model was additionally evaluated using the pooled OOF predictions. The model showed a BS of 0.1036, a CAI of −0.1034, and a CAS of 0.9806 (**Table 7**). As shown in **Figure 4**, the pooled OOF calibration curve generally followed the ideal diagonal reference, although some deviations were observed in individual folds and within intermediate predicted-probability ranges. The calibration slope close to 1 indicated that the overall spread of the predicted probabilities was well preserved.

**Table 7:** Comparison of performance for previously diagnosed DM classification across various classification models.

Model	ID	AUROC (95% CI)	AP (95% CI)	BS	CAI	CAS
ExtraTrees	210	0.8336 (0.8138, 0.8523)	0.6754 (0.6395, 0.7092)	0.1036	-0.1034	0.9806
RF	140	0.8243 (0.8048, 0.8430)	0.6710 (0.6353, 0.7057)	0.1101	0.6016	1.5496
LightGBM	230	0.8048 (0.7808, 0.8257)	0.6703 (0.6343, 0.7064)	0.1123	-0.2216	0.4923
XGBoost	290	0.8012 (0.7781, 0.8237)	0.6619 (0.6245, 0.6958)	0.1147	-0.4489	0.7024
ELR	200	0.6644 (0.6381, 0.6896)	0.3370 (0.3050, 0.3763)	0.2237	-1.3197	0.5463

**Figure 3:**
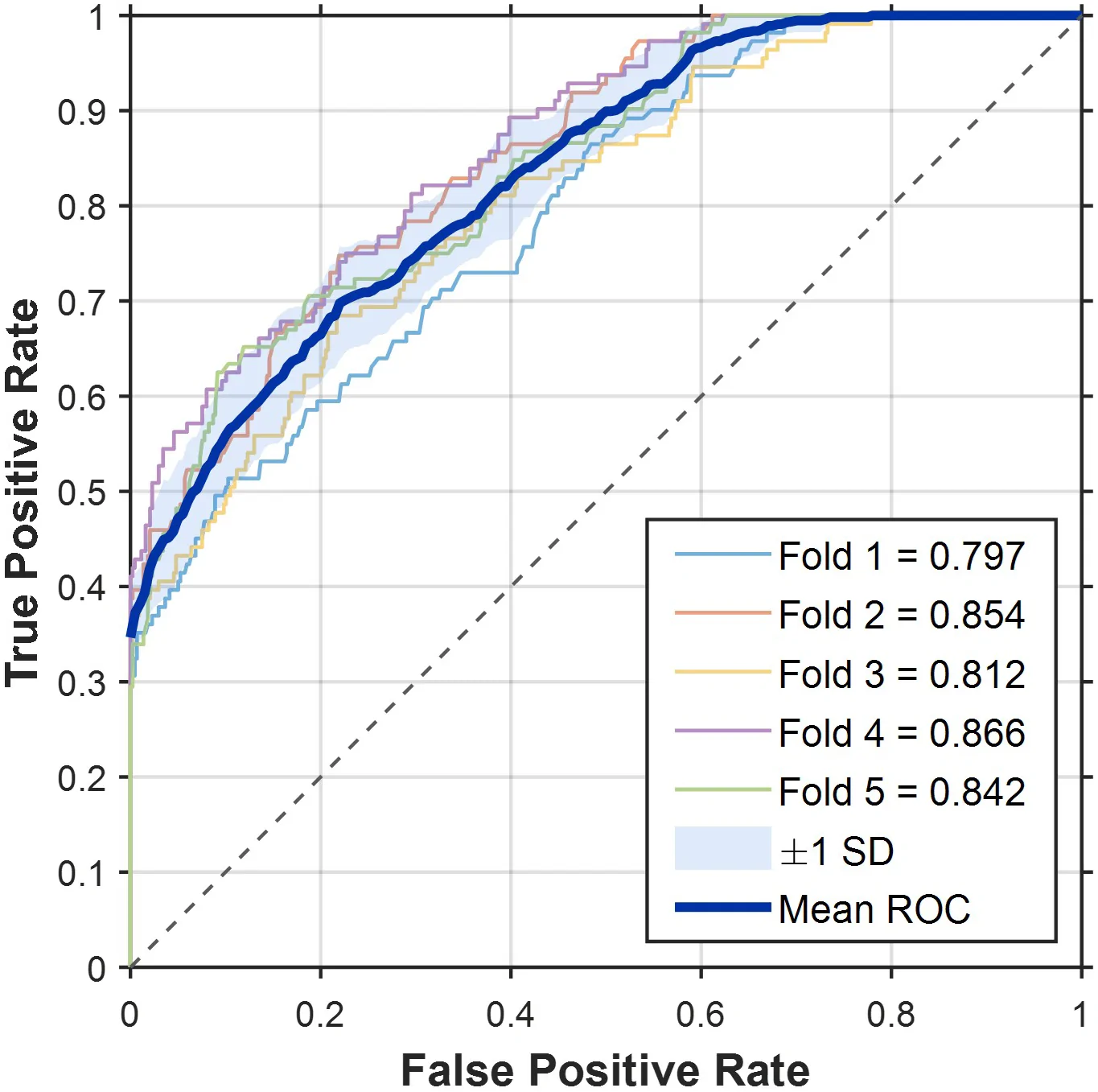
ROC curve of the ExtraTrees model for discrimination of previously diagnosed DM status.

**Figure 4:**
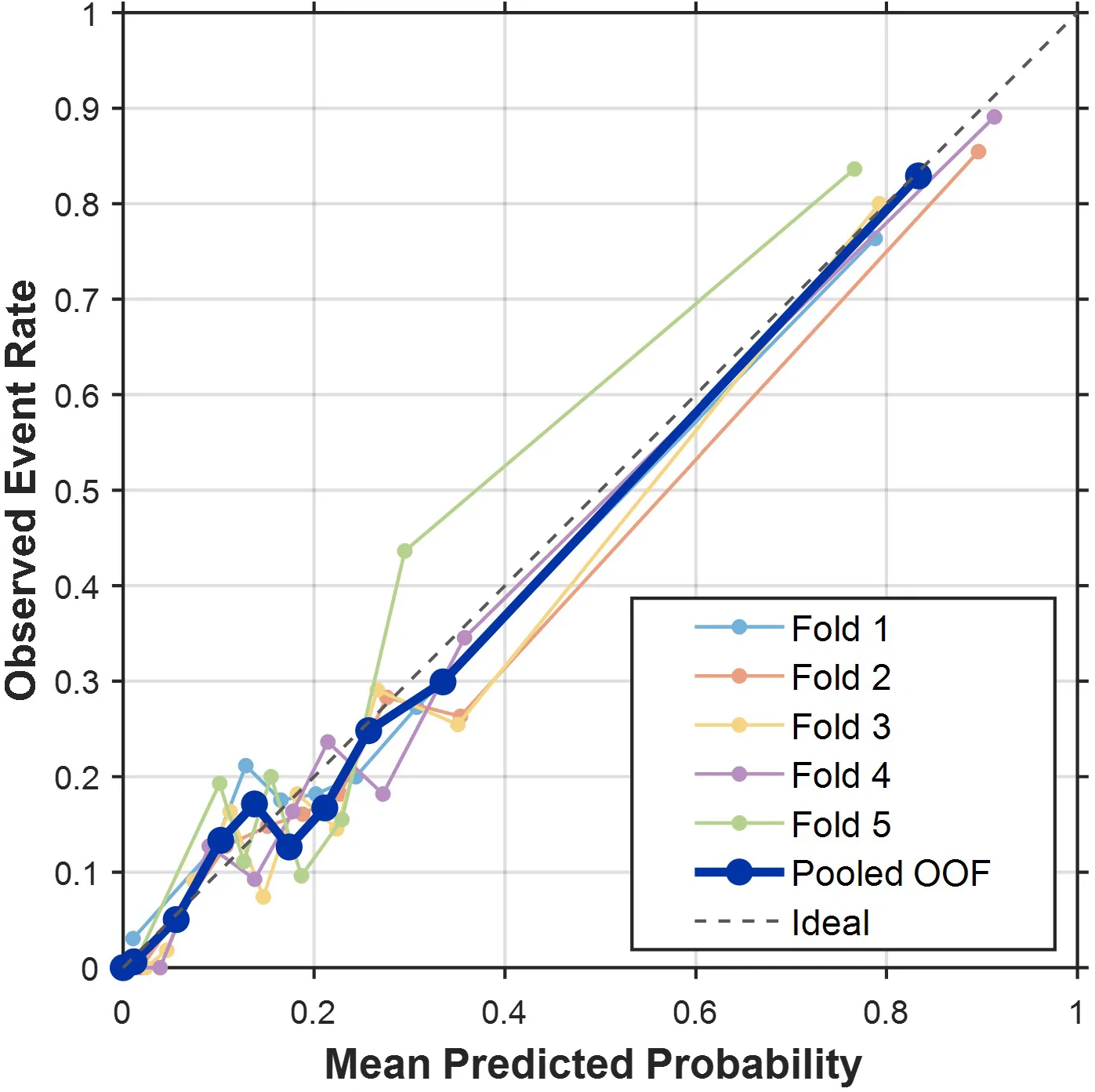
Calibration curve of the ExtraTrees model for classification of previously diagnosed DM status.

When a default threshold of 0.5 was applied, the model exhibited a sensitivity of 0.3627 and a specificity of 0.9945, indicating a conservative classification behavior that prioritized minimizing false positives (**Table 8**). At this threshold, the precision was also very high (0.9439), suggesting that participants classified as positive were highly likely to belong to the DM-positive group. To evaluate variations in performance across different decision thresholds, the threshold was optimized based on BACC and F1-score. The thresholds maximizing BACC and F1-score were determined exclusively from the corresponding inner-CV predictions and were subsequently applied to the held-out outer-test data. This adjustment enhanced sensitivity, leading to a more balanced classification performance, albeit at the cost of reduced specificity and precision as a trade-off. When optimized based on BACC, sensitivity increased to 0.6804, while specificity and precision decreased to 0.7683 and 0.4278, respectively. When optimized based on F1-score, the model showed a sensitivity of 0.5530, specificity of 0.8921, and precision of 0.5662. The corresponding BACC values were 0.7244 and 0.7226 for the BACC- and F1-optimized thresholds, respectively, while the F1-scores were 0.5253 and 0.5595. These results indicate that threshold adjustment substantially increased sensitivity relative to the default threshold, although this improvement was accompanied by reductions in specificity and precision.

**Table 8:** Performance of the ExtraTrees classifier across different decision thresholds.

Threshold	BACC	F1-score	Precision	Sensitivity	Specificity
0.5	0.6787	0.5240	0.9439	0.3627	0.9945
Max BACC	0.7244	0.5253	0.4278	0.6804	0.7683
Max F1	0.7226	0.5595	0.5662	0.5530	0.8921

### Classifier sensitivity analysis

**Table 7** presents the performance of various classifiers, with feature ranking, input dimensionality selection, and hyperparameter optimization performed independently for each model. Among the evaluated classifiers, ExtraTrees demonstrated the highest AUROC of 0.8336 (95% CI, 0.8138–0.8523) and an AP of 0.6754 (95% CI, 0.6395–0.7092). Following this, RF achieved the second-highest performance, yielding an AUROC of 0.8243 and an AP of 0.6710. When utilizing LightGBM, the AP was comparable to that of RF, at 0.6703, but the AUROC showed a more pronounced decline to 0.8048. XGBoost showed slightly lower discrimination, with an AUROC of 0.8012 and an AP of 0.6619. Overall, the four tree-based ensemble models substantially outperformed ELR, which showed the lowest performance, with an AUROC of 0.6644 and an AP of 0.3370. Calibration performance also differed across the classifiers. ExtraTrees showed the lowest BS (0.1036) and a CAS closest to 1 (0.9806). RF showed a BS of 0.1101, a CAI of 0.6016, and a CAS of 1.5496. LightGBM and XGBoost showed BS of 0.1123 and 0.1147 and CAS of 0.4923 and 0.7024, respectively. ELR showed the least favorable overall performance, with a BS of 0.2237, a CAI of −1.3197, and a CAS of 0.5463. Taken together, ExtraTrees showed the most favorable overall balance between discrimination and calibration among the evaluated classifiers.

## Discussion

This study systematically characterized multifrequency segmental BIA responses using a structured high-dimensional descriptor framework for discrimination of previously diagnosed DM status in a predominantly older-adult cohort. Highly ranked descriptors showed substantial cross-fold reproducibility in feature inclusion, although their exact relative ordering varied across folds. The newly developed *D_f_* and *D_r_* descriptors were repeatedly retained across outer-fold feature selection, indicating reproducible participation in the multivariable representation. At the group level, however, their aggregate representation among the selected features and their SHAP contribution did not indicate preferential selection or disproportionate SHAP contribution relative to their representation in the candidate descriptor pool, and their incremental improvement in discrimination was modest. The highly ranked descriptors also reflected multiple bioelectrical dimensions, while the newly developed descriptors were predominantly associated with trunk-based and higher-frequency impedance-response patterns. Taken together, these findings suggest that multifrequency segmental BIA contains distributed predictive information related to DM-associated bioelectrical patterns, with the newly developed descriptors contributing complementary rather than dominant predictive information.

The feature-selection results indicate that dimensionality reduction did not simply produce unrelated fold-specific feature sets. Among the descriptors with the highest global SHAP-based importance, most were repeatedly retained across independently developed outer-training subsets, including many of the highly ranked *D_f_* and *D_r_* descriptors. At the same time, variability in feature-selection ranks showed that the relative ordering of individual descriptors was less stable than their inclusion. This distinction is important because it suggests that the model relied on a reproducible multivariable representation rather than on a small set of descriptors with fixed importance rankings. Given the substantial correlation and partial redundancy expected among BIA-derived variables, such stability at the level of feature inclusion may be more informative than requiring identical rank ordering across folds.

The anatomical and frequency-related distribution of the highly ranked descriptors provides additional insight into the type of bioelectrical information captured by the structured representation. Among the highest-ranked *D_f_* and *D_r_* descriptors, trunk-derived features were predominant, and many were associated with impedance-response changes in the 250–1,000 kHz range. Interval-specific slopes and impedance drops, including their reliability-weighted forms, also appeared repeatedly. From a bioelectrical perspective, current flow at lower frequencies is more strongly constrained by the capacitive properties of cell membranes and therefore predominantly follows extracellular conductive pathways, whereas increasing frequency progressively reduces this capacitive barrier and allows broader conductive pathways, including intracellular pathways, to contribute to the measured impedance response (Kyle et al., 2004; Khalil et al., 2014). Accordingly, the repeated appearance of higher-frequency slope and drop descriptors suggests that the shape and consistency of the frequency-dependent impedance response may capture information related to tissue composition, fluid distribution, and cellular electrical properties beyond absolute impedance magnitude alone. The predominance of trunk-derived descriptors further suggests that central-body impedance patterns may provide useful complementary information, although the present results do not establish a specific anatomical mechanism underlying this pattern. These interval-based descriptors also complement the Cole and impedance-geometry descriptors included in *D_b_*. Cole-type descriptors characterize frequency-dependent behavior through resistance–reactance relationships on the *R*–*X* plane, whereas *D_f_* and *D_r_* summarize local changes in impedance magnitude across six measured frequencies. Because reactance was available at only three frequencies in the present dataset, the interval-based magnitude descriptors provide an alternative representation of finer changes across the available frequency range. However, these associations should not be interpreted as direct physiological identification of extracellular or intracellular fluid compartments. *D_f_* and *D_r_* were derived from impedance magnitude rather than from separately resolved resistance and reactance components, and the residuals incorporated into *D_r_* may reflect physiological variation, measurement variability, or device- and protocol-specific effects. Thus, these descriptors are best interpreted as engineered summaries of frequency-response patterns rather than as direct biomarkers of a specific tissue or fluid compartment.

The inclusion of *D_f_* and *D_r_* resulted in a modest additional improvement in classification performance compared with the configuration using *D_d_* + *D_s_* + *D_b_*. Notably, the addition of *D_r_* alone produced little improvement over the base descriptor configuration, whereas the full configuration including both *D_f_* and *D_r_* yielded the highest point estimates of AUROC and AP. However, the paired comparison with the *D_f_*-containing configuration showed only a small difference with confidence intervals including zero, indicating that the additional contribution of *D_r_* should be interpreted cautiously. Importantly, the same general performance pattern was maintained when the selected dimensionality was fixed at 250 features, with the full descriptor configuration again yielding the highest point estimates, although the absolute differences remained small. This suggests that the observed incremental differences were not solely attributable to variation in the number of selected features. In addition, *D_f_* and *D_r_* together accounted for approximately 39.4% of the fold-specific selected feature sets. However, these groups comprised 38.3% of the original candidate descriptor pool, indicating that their aggregate representation among selected features was broadly proportional to their availability rather than preferentially enriched. Their pooled OOF SHAP contribution was 35.1%, which likewise did not suggest disproportionate group-level predictive importance relative to their representation in the descriptor pool. Taken together, these findings indicate reproducible participation of *D_f_* and *D_r_* descriptors in the multivariable prediction structure, while their aggregate selection and SHAP proportions did not indicate preferential enrichment or disproportionate group-level importance. However, the absolute performance gain associated with their inclusion remained small, and their contribution should therefore be interpreted as complementary predictive information rather than as evidence of a substantial improvement in discrimination.

The DM-positive group accounted for 20.29% of the study population. Because participants were not selected or matched according to DM status, the dataset retained the case distribution of the source cohort rather than an artificially balanced case-control distribution. In addition, no matching according to BMI or other body-composition-related variables was performed, thereby preserving potentially informative anthropometric and bioimpedance variation relevant to DM classification. The DM-positive group also included a higher proportion of men (Ong et al., 2023; American Diabetes Association Professional Practice Committee, 2026a); therefore, the observed differences in height and body weight should be interpreted partly in relation to sex distribution rather than as independent DM-associated characteristics. Accordingly, sex was retained as a demographic input variable because it is related to both anthropometric characteristics and bioimpedance measurements. The additional incorporation of *D_b_*, which systematically structured BIA-derived parameters, further improved classification performance, indicating predictive information beyond that captured by *D_d_* + *D_s_*. These descriptors included PhA-related, Cole model-related, and frequency-response information previously associated with DM or metabolic health (Ward and Brantlov, 2023; Nandwani et al., 2025).

When the full descriptor pool was evaluated using classifier-specific feature-selection and optimization procedures, tree-based ensemble models generally showed favorable performance. ExtraTrees achieved the highest AUROC and also showed the most favorable overall calibration among the evaluated classifiers, whereas RF showed slightly lower discrimination with a comparable AP. LightGBM and XGBoost also retained substantially higher discrimination than ELR. In contrast, ELR showed relatively lower performance, suggesting that the relationship between the selected descriptors and DM status may not be sufficiently explained by a simple linear combination of features. The lower performance of ELR suggests that discrimination may depend partly on nonlinear relationships, interactions, and correlated information across the heterogeneous descriptor set (Darst et al., 2018). Because feature selection and input dimensionality determination were repeated independently for each classifier, these findings should be interpreted as a sensitivity analysis of the descriptor framework to model choice rather than as evidence that a single fixed feature set generalized across algorithms. Beyond discrimination, ExtraTrees also showed favorable overall calibration, although residual systematic calibration error remained. Calibration should therefore be reassessed in independent external populations.

Based on the ExtraTrees classifier, which yielded the highest performance, an imbalance was observed among threshold-dependent metrics, such as precision, sensitivity, and specificity. Specifically, at the default threshold, the model exhibited low sensitivity compared to high precision and specificity. Even when the thresholds optimized for BACC and F1-score within the inner CV were applied to the held-out outer-test data, specificity remained higher than sensitivity. This suggests that the model adopted a conservative classification approach, preferentially identifying only highly certain positive cases rather than aggressively detecting all individuals with DM. The relatively low sensitivity likely reflects multiple factors rather than class imbalance alone. The imbalance between DM-positive and DM-negative participants and decision-threshold selection may have contributed to the observed classification behavior. In addition, overlap between the two groups in the BIA-derived feature space, the use of previously diagnosed DM as the outcome, and treatment-related modification of body composition or fluid distribution may also have reduced the separability of positive and negative cases. More fundamentally, BIA data do not provide a direct measure of DM. While DM status is directly evaluated through blood-based biomarkers such as blood glucose or HbA1c (American Diabetes Association Professional Practice Committee, 2026b), BIA reflects indirect physiological alterations associated with DM, including body composition, fluid distribution, fat mass, muscle mass, and impedance (Ward, 2019; Ward and Brantlov, 2023). Consequently, the model captures body-composition and bioelectrical patterns associated with DM rather than glycemic status itself, and overlap may occur between participants with and without diagnosed DM when similar physiological alterations arise from other metabolic or age-related conditions or are modified by treatment. These characteristics imply a substantial overlap between the DM and non-DM groups within the BIA-based feature space, which may partly contribute to the remaining imbalance between sensitivity and specificity even after threshold optimization. Nevertheless, BIA is a noninvasive, low-cost, and repeatable modality, making the present framework potentially attractive for future screening-support applications (Böhm and Heitmann, 2013). In the present cohort, threshold adjustment demonstrated that the balance between sensitivity and specificity could be modified within the classification task. These findings support the methodological feasibility of further evaluating structured BIA information for future screening-support applications. However, because the model was developed to discriminate previously diagnosed DM rather than blood-test-defined undiagnosed DM, the present operating characteristics should not be interpreted as validated screening performance for an undiagnosed population. If future studies confirm its performance using contemporaneous blood-test-defined outcomes in appropriate screening populations, future work could evaluate whether the framework can help prioritize individuals for confirmatory blood testing.

As noted in the Introduction, previous BIA-based studies on DM have primarily focused on analyzing associations or correlations between specific BIA parameters and DM, whereas studies directly evaluating screening performance have been limited. In this regard, Jun et al. evaluated the feasibility of DM classification based on PhA-related parameters derived from multifrequency BIA and reported AUROC values of 0.827 and 0.845 in men and women, respectively (Jun et al., 2018). The final model in the present study achieved an AUROC of 0.8336, which was within a similar range to the performance reported in that study. The discrimination achieved in the present study was therefore broadly comparable to that reported using the simpler PhA-based approach rather than clearly superior. Accordingly, the principal contribution of the present study should not be interpreted as a substantial increase in predictive performance, but rather as the systematic characterization of multifrequency segmental BIA responses using a broader structured descriptor framework. Direct comparison of these results should nevertheless be made with caution because the two studies differed in terms of dataset characteristics, study design, and validation procedures. For example, the study by Jun et al. used a relatively balanced case-control design in which patients with DM and controls were matched according to age, sex, and BMI, whereas the present study had an imbalanced sample distribution, with DM-positive participants accounting for 20.29% of the total study population. Matching according to BMI was not performed because body-composition-related characteristics may themselves carry predictive information linking DM status to bioimpedance, and matching on such variables could remove informative variation. This design also preserved the naturally imbalanced case distribution of the source cohort. In addition, whereas the previous study included 90 participants, the present study evaluated model performance in a substantially larger cohort of 2,745 participants, which represents a strength of the present study.

Several limitations should be considered when interpreting the present findings. First, DM status was determined from self-reported history of previously diagnosed DM obtained during medical interviews rather than from contemporaneous blood-based diagnostic criteria. Consequently, undiagnosed DM cases may have been included among participants classified as negative, and the present outcome does not directly correspond to the target population for undiagnosed-DM screening. Because diagnosis and self-report may also be related to participant characteristics associated with bioimpedance, the direction and magnitude of bias resulting from outcome misclassification cannot be determined from the present data. In addition, participants with previously diagnosed DM may have received pharmacological treatment or lifestyle interventions that altered body composition or fluid distribution, potentially modifying DM-associated BIA patterns. Because information on diabetes treatment status and disease duration was not available, stratified analyses could not be performed to distinguish treatment-related from disease-related BIA patterns. Second, DM subtype information was not available in the cohort. Therefore, type 1 and 2 DM could not be distinguished, and subtype-specific differences in BIA characteristics or classification performance could not be evaluated. Third, participants were originally recruited through a dementia-related cohort rather than specifically for DM screening. Consequently, selection into the cohort may have been influenced by health characteristics associated with both DM and body composition, cognitive status, or frailty. Such recruitment-related selection may have affected the observed relationships between BIA-derived features and DM status and may limit the transportability of the present findings to a general screening population. **Figure 5** summarizes these structural considerations, including target mismatch, treatment-related pathways, shared factors, cohort selection, and the measurement process. Fourth, BIA reflects body composition, hydration, and tissue electrical properties rather than hyperglycemia directly. The model may therefore partly capture a broader metabolic or frailty-related phenotype associated with previously diagnosed DM. Aging, obesity, sarcopenia, chronic kidney disease, heart failure, and other conditions may produce overlapping alterations in body composition or fluid distribution, potentially affecting the transportability of BIA-based prediction across populations. Fifth, all measurements were obtained using a single BIA device, at a single site, in the supine posture, and under a standardized measurement protocol. In particular, *D_r_* may reflect device- or protocol-specific measurement characteristics in addition to physiological variation, and its robustness across devices, postures, and measurement settings remains to be established. Sixth, model performance was evaluated using internal validation within a single cohort, and no independent external cohort was available. Accordingly, the reported performance does not establish transportability to other populations, institutions, devices, or measurement settings. Seventh, the high-dimensional descriptor space contained potentially correlated and redundant variables, which may affect the stability and relative ranking of selected features. Although many highly ranked *D_f_* and *D_r_* descriptors were repeatedly retained across outer folds, variability remained in their relative feature-selection ranks. However, their incremental improvement in discrimination was modest relative to the additional feature-engineering complexity. Their contribution should therefore be interpreted as complementary predictive information rather than as evidence of a substantial performance advantage. Future studies should evaluate the proposed framework using blood-test-defined outcomes, including undiagnosed DM, in broader and independently recruited populations and should assess its robustness across multiple BIA devices and measurement protocols.

**Figure 5:**
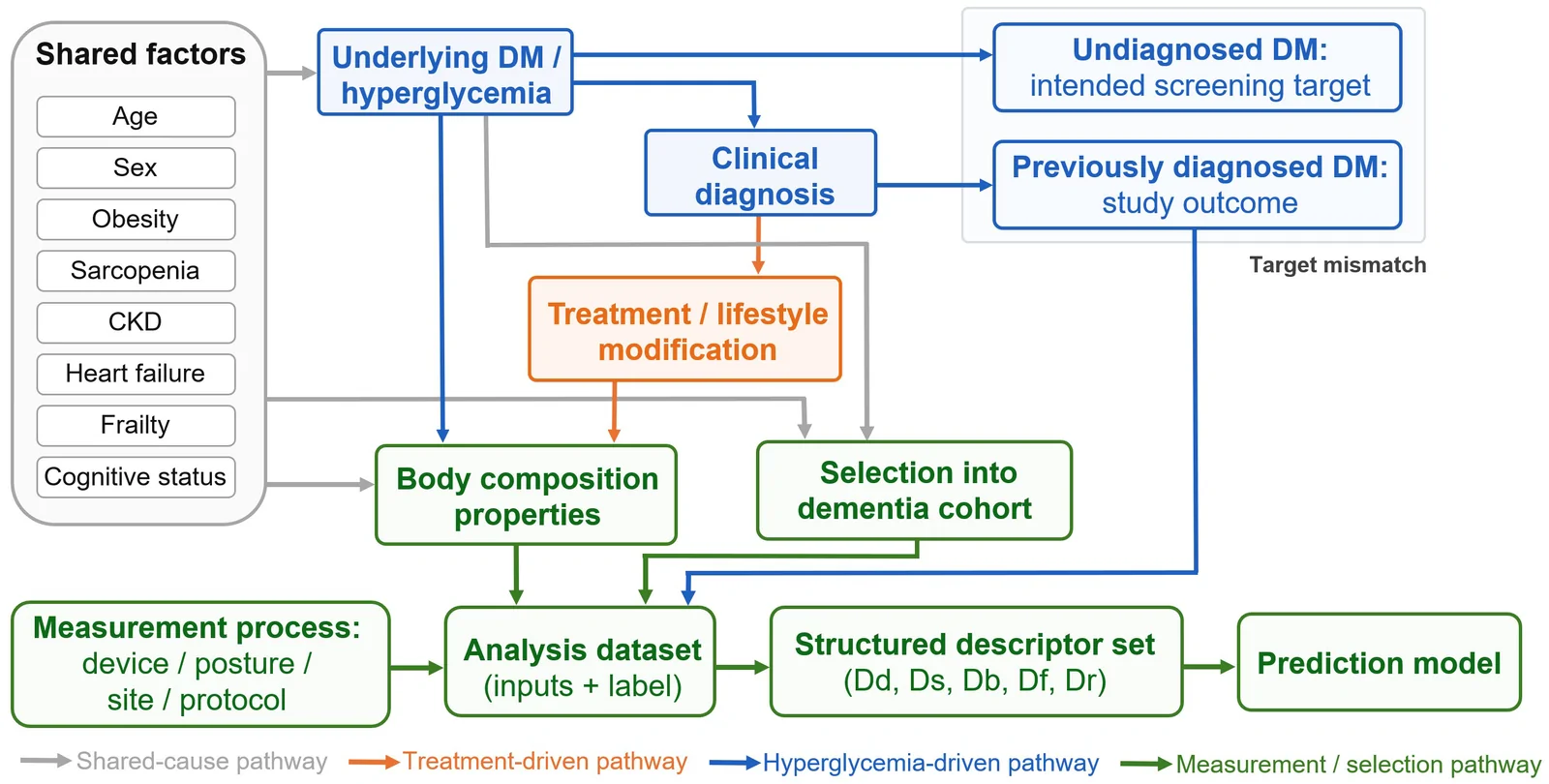
Conceptual causal diagram for BIA-based DM screening support.

## Conclusion

Previous studies have reported associations between BIA-derived parameters and DM. However, studies that quantitatively evaluate DM screening performance based on such information remain limited. To fill this research gap, the present study partially extended and systematically organized previously introduced BIA-derived parameters to develop a multifrequency, segmental BIA descriptor-based framework for DM screening, and it was evaluated using a relatively large dataset of 2,745 participants. As a result, the ExtraTrees-based classification model showed favorable discriminative performance, with an AUROC of 0.8336 and an AP of 0.6754 based on pooled OOF predictions. The full descriptor configuration achieved the highest overall discrimination, although the incremental improvement associated with the newly constructed *D_f_* and *D_r_* was modest. Highly ranked *D_f_* and *D_r_* descriptors were repeatedly retained across outer-fold feature selection, and the two descriptor groups participated in SHAP-based predictive attribution, supporting their role as complementary predictive information rather than as evidence of a substantial performance advantage. The proposed model is not intended to replace conventional diagnostic tests for DM. Rather, the present study demonstrates that structured multifrequency segmental BIA descriptors can provide a reproducible and complementary representation of bioelectrical patterns associated with previously diagnosed DM. However, its ability to identify individuals with undiagnosed DM remains to be established because the present findings were derived from internal validation in a single cohort with DM status defined from previously diagnosed disease rather than contemporaneous blood-test criteria. Accordingly, the present framework provides a methodological basis for future evaluation of BIA-assisted screening in independently recruited populations using contemporaneous blood-test-defined outcomes. Future studies should also assess its robustness across different BIA devices and measurement protocols.

## Data Availability

The raw data supporting the conclusions of this article will be made available by the authors, without undue reservation.
